# Antigen presenting cells in cancer immunity and mediation of immune checkpoint blockade

**DOI:** 10.1007/s10585-023-10257-z

**Published:** 2024-01-23

**Authors:** Cassia Wang, Lee Chen, Doris Fu, Wendi Liu, Anusha Puri, Manolis Kellis, Jiekun Yang

**Affiliations:** 1 Computer Science and Artificial Intelligence Laboratory, Massachusetts Institute of Technology, Cambridge, MA USA; 2 Broad Institute of MIT and Harvard, Cambridge, MA USA; 3 Department of Stem Cell and Regenerative Biology, Harvard University, Cambridge, MA USA; 4 Department of Biological Engineering, Massachusetts Institute of Technology, Cambridge, MA USA

**Keywords:** Cancer immunity, Immune checkpoint blockade, Antigen presenting cells, Dendritic cell, Monocyte, Macrophage

## Abstract

Antigen-presenting cells (APCs) are pivotal mediators of immune responses. Their role has increasingly been spotlighted in the realm of cancer immunology, particularly as our understanding of immunotherapy continues to evolve and improve. There is growing evidence that these cells play a non-trivial role in cancer immunity and have roles dependent on surface markers, growth factors, transcription factors, and their surrounding environment. The main dendritic cell (DC) subsets found in cancer are conventional DCs (cDC1 and cDC2), monocyte-derived DCs (moDC), plasmacytoid DCs (pDC), and mature and regulatory DCs (mregDC). The notable subsets of monocytes and macrophages include classical and non-classical monocytes, macrophages, which demonstrate a continuum from a pro-inflammatory (M1) phenotype to an anti-inflammatory (M2) phenotype, and tumor-associated macrophages (TAMs). Despite their classification in the same cell type, each subset may take on an immune-activating or immunosuppressive phenotype, shaped by factors in the tumor microenvironment (TME). In this review, we introduce the role of DCs, monocytes, and macrophages and recent studies investigating them in the cancer immunity context. Additionally, we review how certain characteristics such as abundance, surface markers, and indirect or direct signaling pathways of DCs and macrophages may influence tumor response to immune checkpoint blockade (ICB) therapy. We also highlight existing knowledge gaps regarding the precise contributions of different myeloid cell subsets in influencing the response to ICB therapy. These findings provide a summary of our current understanding of myeloid cells in mediating cancer immunity and ICB and offer insight into alternative or combination therapies that may enhance the success of ICB in cancers.

## Introduction to myeloid cells

Myeloid cells are present in immunological settings, where they circulate through the blood and lymphatic system and are recruited to sites of tissue damage and infection [1]. In the tumor microenvironment (TME), myeloid cells can either be immune-activating and/or suppressing. Efforts have been made to target suppressive myeloid cells in cancer, such as tumor-associated macrophages (TAMs), polymorphonuclear myeloid-derived suppressor cells (PMN-MDSCs) from the neutrophil lineage, mononuclear myeloid-derived suppressor cells (M-MDSCs) from the monocyte lineage, and a combination of myeloid cells [2]. However, research in the role of myeloid cells in cancer, particularly cancer immunity, was generally lacking compared to lymphoid cells. Recently, there has been a heightened focus on myeloid cells due in part to advances in single-cell technology [3]. Here, we present a succinct overview of the general myeloid cell classes as well as their developmental origins and functions.

**Fig. 1:**
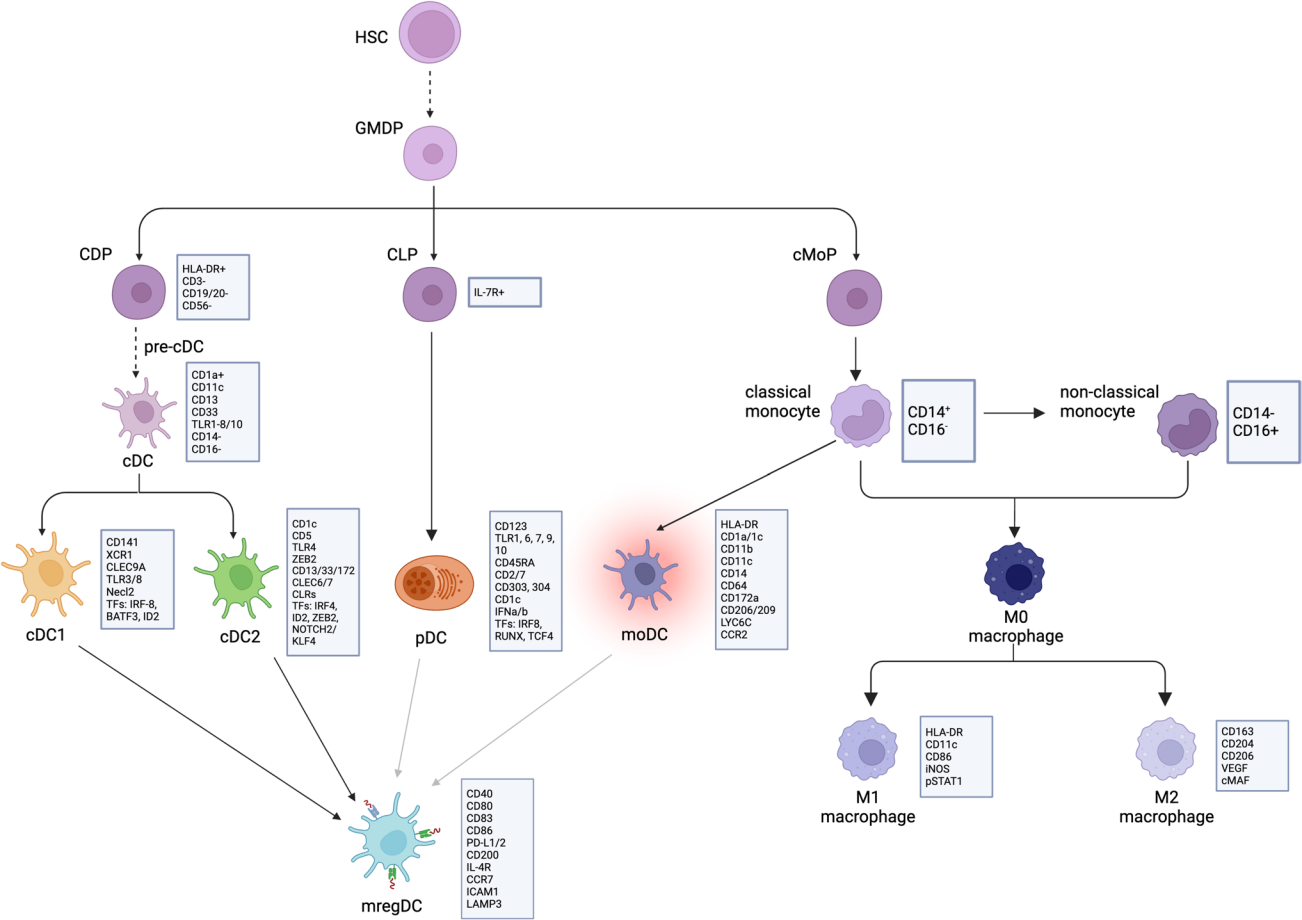
Ontogeny of dendritic cell and monocyte lineages (starting from hematopoietic stem cells) along with well-identified surface markers, transcription factors, and necessary growth factors for each class. Starting from hematopoietic stem cells (HSC), human myeloid cells are derived from the granulocyte–macrophage DC progenitor (GMDP) branch into common DC progenitors (CDP) and common monocyte progenitors (cMoP), giving rise to pre-conventional DCs (pre-cDC) and monocytes, respectively. Pre-cDCs finally differentiate into cDC1 and cDC2. Monocyte-derived DCs (moDCs) can differentiate from monocytes in the presence of inflammatory conditions. Previously, plasmacytoid DCs (pDCs) were thought to also come from the CDP lineage with cDCs, but higher resolution analysis shows pDCs arising from the common lymphoid progenitor (CLP), which also begins as HSCs [13]. Unlike cDCs and moDCs, pDCs are not present as non-lymphoid cells [14]. Monocytes and macrophages are derived from common monocyte progenitors (cMoP). cMoPs differentiate into classical monocytes, from which they may transition into an intermediate or non-classical phenotype [95]. Classical monocytes may also differentiate into moDCs. All subsets of monocytes may develop into M0 macrophages, which further delineate into M1 and M2 macrophages. These subsets may be referred to tumor-associated macrophages (TAMs) within the tumor microenvironment [96]. *Additional sources* [4, 8, 10, 29, 30, 43, 44, 97]. Created with BioRender.com

DCs are key professional antigen-presenting cells (APCs) in the immune system alongside macrophages [4] and are the “primary investigators” of adaptive immunity, presenting antigens to T cells [5]. DCs are needed to maintain tissue homeostasis in steady-state conditions or mount an antigen-specific T cell response by sensing danger signals in inflammatory conditions [6]. Named after their branching phenotype, DCs present endogenous or exogenous antigen peptides on their surface via the major histocompatibility complex (MHC) I or II [6, 7]. DCs are activated by sensing environmental signals such as cytokines, pathogen-associated molecular patterns (PAMPs), and damage-associated molecular patterns (DAMPs) on their receptors [8]. At the time of an inflammatory response, DCs pass through the process of “maturation” where the expression of MHC class I and II and co-stimulatory molecules are upregulated. The resulting mature and immunogenic DCs migrate to the lymph node where they encounter T cells [6, 7, 9]. T cells are only fully activated via certain signaling interactions with APCs. The ontogeny of DCs has been redefined several times and continues to advance as single cell technology allows for the detection of the dynamic development of cells and detection of transcriptional profiles on minimal subsets, which was reviewed in other places [3, 10–13]. Initially, it was thought that the precursors of DCs were epidermal Langerhans cells [11]; now, it is known that the origin of DCs begins with the development of hematopoietic stem cells (HSCs) and is derived from the bone marrow [10, 11]. Granulocytes, monocytes, and DCs share a common progenitor downstream of HSC termed granulocyte–macrophage DC progenitors (GMDPs), from which multiple progenitors of combinations of the three classes further differentiate into, which is summarized in Fig. 1 [14].

Monocytes and macrophages function as key mediators of the innate immune response. Monocytes are classified as either classical monocytes, marked by CD14^+^ and CD16^−^, or non-classical monocytes, marked by CD14^−^ and CD16^+^ [15]. Generally, macrophages are central to the phagocytosis of cellular debris, production of proinflammatory cytokines, and presentation of antigens to T cells, thus playing a crucial role in both the innate and adaptive immune system [16]. In the ontogeny of macrophages, GMDPs were originally thought to be the sole cells capable of differentiating into macrophages [17, 18]. However, we now know that most macrophages differentiate from embryonic precursors, with a lesser proportion hailing from GMDPs and tissue-resident monocyte precursors [18, 19]. Despite the influence of their microenvironment on macrophage gene expression, embryonic precursor derived macrophages remain distinct [20]. The functional differences between embryonic precursor-derived and GMDP-derived macrophages is an active field of research and is not yet rigorously investigated [20–22]. The migration, differentiation, and function of monocytes in the TME have been thoroughly reviewed in other literature [23–25]. The ontogeny of monocytes and macrophages is visualized in Fig. 1.

## Dissecting myeloid cell subsets in cancer immunity

Myeloid cell subsets play a diverse set of roles in mediating tumor migration, progression, angiogenesis, and metastasis [26, 27]. In this review, we focus on their distinct roles in mediating cancer immunity.

### Conventional, monocyte-derived and plasmacytoid dendritic cells

A number of reviews have attempted to classify DC subsets based on differentiating markers as well as trace their roles in cancer [4, 8, 26–30]. Although the classification of DCs is ongoing and context-dependent [28], subsets are largely conserved across human solid cancers [31]. In the TME, DCs present tumor-associated antigens (TAAs) or neoantigens expressing the genetic alterations of malignant cells [8, 32]. After undergoing maturation, DCs migrate to the tumor-draining lymph node (tdLN) where the T cells are primed and activated [28, 33]. Although the role of DCs reflects a pro-inflammatory nature, studies have also discussed the potential of tolerogenic dendritic cells, not only in the context of autoimmunity but also recently in cancer [34, 35]. The contribution of each subset of DCs in facilitating pro-tumor or anti-tumor immunity depends upon the markers they express and the cytokines they produce.

Conventional dendritic cells (cDCs) develop from CDPs in the bone marrow and differentiate into the subsets cDC1 and cDC2 which both have resident and migratory populations [28]. cDC1s are characterized by the expression of CD141^+^ in humans and produce a strong immune response by cross-presenting intracellular pathogens from necrotic and apoptotic tumor cells on MHC I molecules to CD8^+^ T cells. It is suggested that they may also prime CD4+ T cells [4, 8]. Circulating cDC1s may be recruited by natural killer (NK) cells which produce chemo-attractants and lead to an accumulation of cDC1s in the TME [4, 28]. There, cDC1 infiltration may be limited by tumors with active β-catenin via CCL4 expression [30]. The immunogenicity [36] and clonality [37] of tumors also plays a role in the effect of cDC1. Migratory CD103^+^ cDC1s cross-prime tdLN-resident naive CD8^+^ T-cells in a CCR7-dependent manner (see *Migratory and regulatory dendritic cells*) but may also transfer antigens to resident DCs within the tdLN [28]. It was also found that cDC1s may maintain a reservoir of tumor-antigen specific TCF1^+^ CD8^+^ T cells in the tdLN in a localized model of lung adenocarcinoma [31]. cDC1s contribute to anti-tumor immunity via production of IL-12, as well as secretion of type I and III interferon (IFN) and expression of IRF8, but tumors may suppress IRF8 dependent development of cDC1s, limiting their antitumor effects [28].

cDC2s are more heterogeneous than cDC1s but are characterized by the expression of the CD1c (BDCA1) surface marker. cDC2s help with host barrier protection and present antigens from extracellular pathogens to CD4^+^ T cells [4, 28] but are also capable of cross-presenting to CD8^+^ T cells [33]. CD1c^+^ cDC2s may also be restricted by regulatory T cells (Tregs) in their ability to recruit CD4^+^ T-cells to the tumor [38]. Overall, cDC2 contributes to anti-tumor immunity by TAA presentation to CD4^+^ T cells or the transfer of TAAs to lymphoid tissue-resident DCs but is dependent on cDC1 function and the presence of Tregs [28, 33]. cDC2s may rely on interferon stimulation to produce an anti-tumor immune response. Some studies in mice and humans suggested cDC2s may also play a pro-tumor/immunosuppressive role by preventing CD4^+^ effector T cell function or correlating with Tregs and an exhausted T cell population [38]. Two subsets of cDC2s, DC2 and DC3, have been distinguished based on the expression of CD5. CD5^−^ DC3s are not to be confused with mature and regulatory DCs (mregDCs) or moDCs (discussed later) as some studies have termed them as DC3 [4, 26]. More cDC2-specific information is detailed in Binnewies et al*.* [38] and Saito et al. [33].

moDCs are thought to arise from monocyte DC progenitors [4] and are recruited to the TME by the CCR2-CCL2 chemokine signaling axis [28] (which also recruits monocytes and MDSCs) and is known to favor cancer development [39]. moDCs stimulate Tregs but may also confer anti-tumor immunity by stimulating the helper T cells Th1, Th2 and Th17, and CD8^+^ T cells [8, 28]. moDCs may be seen in specific inflammatory contexts and have a minor role in migration and activity in secondary lymphoid organs [6]. moDCs may not be as effective in stimulating T cells as cDCs. cDC2s and moDCs share several markers, making it difficult to differentiate between them in inflammatory conditions, where moDCs’ function is activated [29, 30].

pDCs arise from the lymphoid lineage and are major producers of type I interferons (IFNs). pDCs are poor at priming naive T cells but can be stimulated to activate CD8^+^ T cells [30]. Though pDCs normally aid in host defense of viral infections and tumors via expression of certain pattern recognition receptors (PRRs), their function is predominantly tolerogenic in a malignant context. pDC IFN-⍺ production is impaired by TME factors such as IL-10 and TGF-β, which are immunosuppressive [4, 8]. A study investigating pDC function also identified TNF-⍺ to be rich in HPV-negative tumors along with IL-10, contributing to an immunosuppressive environment and a decrease in pDC’s tumor-infiltrating capacity [40]. Higher pDC frequencies in the tumor are associated with overall worse prognosis and survival, but pDCs may be beneficial in circulation [4, 28]. The function of pDCs has also been characterized in more detail in Fu et al. [41].

### Mature and immunoregulatory dendritic cells

The last subset of DCs we discuss are mregDCs, which are DCs in an activated state that have been characterized mostly in cancer-specific contexts. mregDCs mainly evolve from the cDC subsets but may also come from moDCs and pDCs. Despite mregDCs being distinguished by their absence of key markers commonly found in other subsets, thereby classifying them as a unique group, the process of sorting other subsets based on canonical markers might incorporate mregDC populations. Thus, findings in the other subsets may also be relevant to mregDCs. mregDCs display a multitude of functions with a considerable amount of heterogeneity existing within the population across cancers [28, 42].

**Fig. 2:**
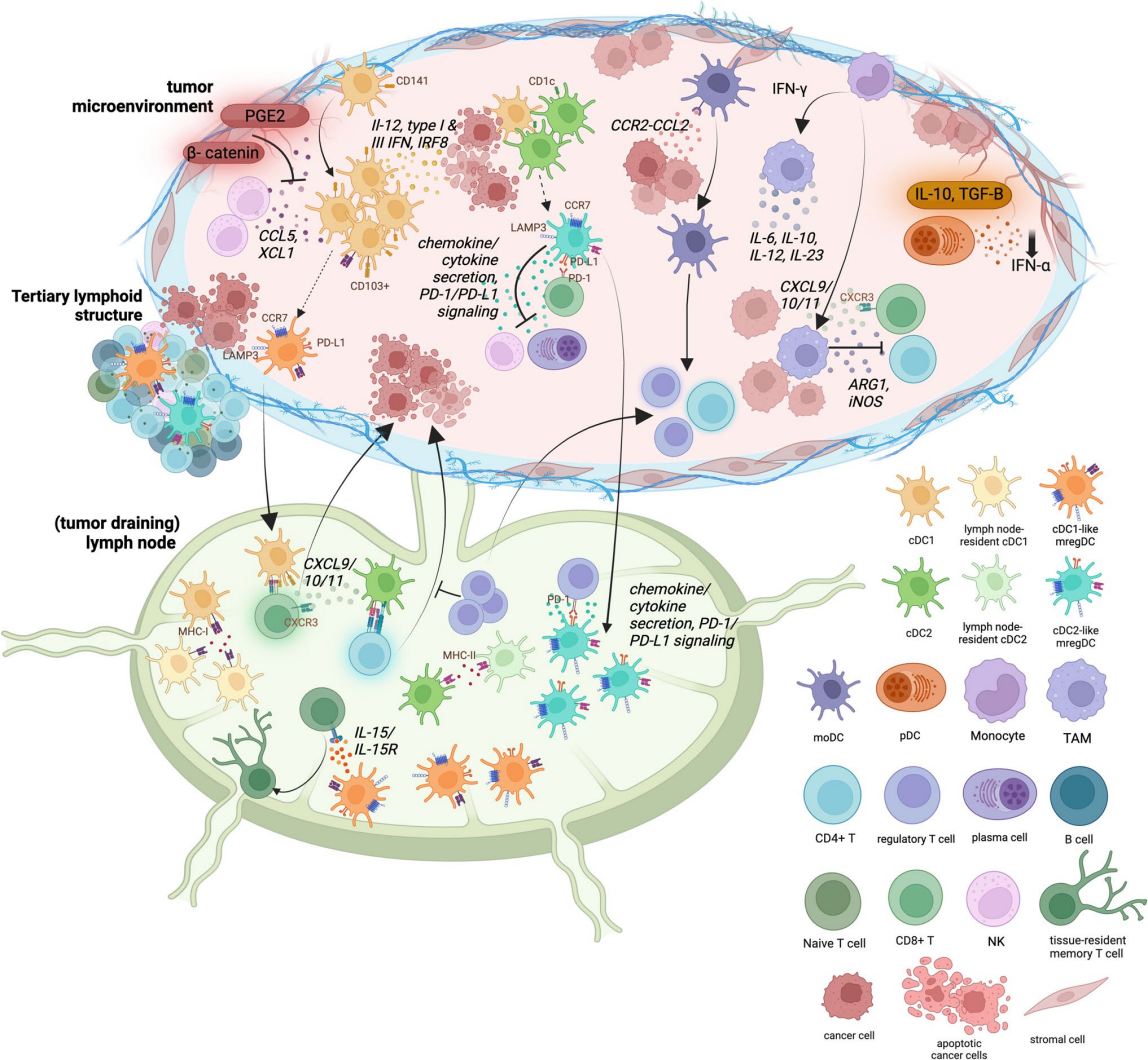
General overview of the interactions between immune cells in the tumor microenvironment, highlighting the functions of the major dendritic cell subsets along with monocytes and tumor-associated macrophages.. Subset-specific markers shown on cells; CD141^+^ and CD1c^+^ used to identify cDC1s and cDC2s, respectively. cDC1 and cDC1-like mregDCs show a greater anti-tumor immunity profile than other dendritic cell (DC) subsets. β-catenin inhibits cDC1 infiltration, and prostaglandin E_2_ (PGE2) may limit interaction of natural killer (NK) cells and cDC1. Migratory cDC1 are characterized by CD103^+^ expression. cDC1s and cDC2s present antigens via their MHC molecules to T cell receptors. Migratory cDC1s and cDC2s present antigens to other lymph node-resident cDC1 and cDC2. cDC2, cDC2-like mregDCs, and moDCs interact with regulatory T cells (Tregs). mregDCs are identified by expression of LAMP3 along with CCR7 and PD-L1. LAMP3^+^ mregDCs are more enriched in tumor-draining lymph nodes. mregDCs may form tertiary lymphoid structures (whether TLS-resident mregDCs are cDC1-like versus cDC2-like has not been studied). cDC2-like mregDCs may inhibit NK, CD8^+^ T, and plasma cells. moDCs are recruited to the TME by the CCR2-CCL2 signaling axis and may stimulate CD4^+^ T cells. pDC production of IFN-⍺ is suppressed in the TME by factors produced by the tumor such as IL-10 and TGF-β. Monocytes migrate to the TME and are stimulated by IFN-γ to differentiate into TAMs. TAMs secrete various levels of IL-6, IL-10, IL-12, and IL-23 based on their polarization. Additionally, TAMs secrete ARG1 and iNOS to inhibit CD4^+^ and CD8^+^ T cell activity. Generally, interactions between myeloid cells and T cells such as CXCL9/10/11 and CXCR3 influence the fate of T cell function [98, 99]. Important biomarkers and functional markers are labeled and colored in brown. Cytokines and chemokines are matched to the cell type that produces them by color and are labeled in black italics. Created with BioRender.com. *Sources* [4, 28–30, 33, 38, 42]

mregDCs have been largely identified by expression of LAMP3, CCR7, and PD-L1, which are responsible for exogenous antigen presentation, migration from primary tumors to lymph nodes, and immune suppression, respectively [4, 26, 28, 42]. mregDCs release an increased amount of cytokines and chemokines relative to immature DCs and express the highest levels of immune checkpoint transcripts out of the DC subsets. mregDCs form tertiary lymphoid structures (TLS) with T and B cells [4, 32, 42] which sustain an immune response and contribute positively to patient survival. Li et al. [42] presents the interaction of specific molecules with mregDCs which have been found through scRNA-seq studies. These interactions are also visually represented in Fig. 2.

Interestingly, mregDCs exhibit both anti- and pro-tumorigenic properties. mregDCs promote antitumor activity by the expression of costimulatory molecules to activate T cells as well as interact with B cells and NK cells. Tumor-infiltrating mregDCs seem to be derived from cDC1s, whereas cDC2-like mregDCs show an expression profile that leans toward an immunosuppressive function. cDC2-like mregDCs promote immune tolerance by the release of specific cytokines and chemokines such as CCL17, CCL19, CCL22, IL-10, IL-4, and IL-35, which can promote Treg migration into the TME, inhibit CD8^+^ T-cell proliferation and effector function, and/or inhibit NK and plasma cell proliferation. More features of these cDC1 and cDC2-like mregDCs and related studies are detailed in Li et al. [42].

With regards to prognostic value, mregDCs generally positively contribute to overall survival and better clinical outcomes [4]. Numerous studies showing a positive correlation between LAMP3^+^ DCs density and better outcome in a variety of cancers imply the prognostic value of mregDCs, although mregDCs are still continuing to be defined. So far, little evidence shows the immunosuppressive role of mregDCs discussed earlier contributing negatively to prognosis, but mregDCs’ role in pro-tumor immunity may be masked by the heterogeneity of the subset and how they are identified in various studies [42].

### Macrophage subsets

Macrophages are highly plastic and polarize themselves toward pro-inflammatory or anti-inflammatory phenotypes. In the context of tumors, their activity is heavily influenced by the TME and tumor immune microenvironment (TIME) and make up the greatest proportion of immune cells in the TME. Pro-inflammatory, or M1 macrophages, play crucial roles in phagocytosis, antigen presentation, and immune regulation. M1 macrophages are induced by IFN-γ and express IL-6, IL-12, IL-23, and IL-10 (low) [43, 44]. Anti-inflammatory macrophages, or M2 macrophages, generally have functions in tissue growth and repair. M2 macrophages are activated by a variety of signals including IL-4, IL-10, and IL-13 and can be identified by their expression of IL-6, IL-10, IL-12, and IL-23 (low) [43, 44]. It is essential to acknowledge that the polarization of macrophages exists on a functional spectrum, resulting in an overlap of expressed interleukins and a corresponding difficulty to classify macrophages into the M1 or M2 phenotype [43, 44].

Tumor-associated macrophages (TAMs) are macrophages found in the tumor that exhibit a pro-tumor, immunosuppressive phenotype and contribute to tumor progression and metastasis. They suppress the activity of the adaptive immune system by expressing immune checkpoint molecules such as programmed death ligand-1 (PD-L1), preventing their elimination. They also produce ARG1, iNOS, and other immune checkpoints to inhibit T cell activity and also serve as markers for their identification in single-cell mRNA sequencing (scRNA-seq). Moreover, TAMs display a high degree of heterogeneity both morphologically and functionally. This heterogeneity is influenced by the TME and enables them to adapt to various conditions and fulfill different roles. Additionally, an inverse correlation has been shown between macrophage recruitment and T cell TME infiltration. Thus, there is a growing interest in engineering macrophages to recognize TAMs and other tumor growth promoters by modifying surface receptors to specifically target TAMs and other tumor growth promoters [44, 45].

scRNA-seq analyses reveal more information about macrophage polarization than the traditional in vitro description of M1 and M2 macrophage polarization. In a pan-cancer study conducted by Cheng et al. [46], the transcriptional profiles of monocytes and macrophages were heavily influenced by the TME, consistent with previous studies. Instead of classification using the traditional M1/M2 polarization, they classified macrophages based on unbiased clustering of single-cell transcriptomes and gene markers for each of the clusters. They identified groups such as C1QC^+^ macrophages present across most cancer types, and IL-1β^+^ macrophages present only in kidney cancer. Using their classification and gene programs linked with angiogenesis, they found that SPP1^+^ and angiogenesis-associated macrophages were linked with poor prognosis [46].

## Role of myeloid cells in immune checkpoint blockade (ICB)

Given the direct role of DCs in the priming and activation of T-cells and the influence of varying T-cell subsets on macrophage polarization and MDSC recruitment, understanding the underlying causes and mechanisms of these interactions has become critical in deciphering ICB efficacy. It has been shown that intratumoral DC and macrophage abundance and/or function may influence tumor response to immunotherapy [28, 45]. Here, we discuss recent studies highlighting key markers and features of DCs and macrophages that offer prognostic value specifically in immune checkpoint blockade (ICB) therapy, which targets immune checkpoint molecules that are expressed on the surface of various immune cells. First, we discuss the direct targets of ICB therapy, such as PD-1, PD-L1, and CTLA-4, on DCs and macrophages. We also discuss some surface markers of specific DC subsets that hold prognostic value in ICB-treated tumors, and the myeloid-cell-specific checkpoint CD47. Finally, we discuss the findings of several signaling pathways occurring upstream or downstream of DC and T cell interaction, and the potential for anti-CD47 and anti-PD-1 combination therapy, which may provide additional insight in the modulation of a successful ICB response. These findings (excluding studies from reviews) are visualized in Fig. 2 and summarized in Table 1 for DCs and Table 2 for macrophages**.**

**Table 1:** Significant findings associated with the impact of dendritic cell (DC) features in immune checkpoint blockade (ICB)-treated models from various studies

DC subset	Feature/marker of interest (on DC)	Model	Treatment	Findings	Implication	Study
Tumor-infiltrating cDC1	PD-L1	DC-conditional PD-L1 knockout B6 mice with MC38 tumor	Anti-mouse PD-L1	No therapeutic effect of PD-L1 blockade therapy in DC-conditional PD-L1 knockout mice (PD-L1 levels remain the same on other cells). Specifically, cDC1 depleted mice did not respond to anti-PD-L1 treatment	PD-L1 on DCs, specifically cDC1s, is crucial for the efficacy of anti-PD-L1 treatment	Peng et al. [48]
moDC	CTLA-4	moDCs cultured with human colorectal cancer cell lysate	CTLA-4 siRNA	CTLA-4-silenced DCs shows enhanced TNF-α, decreased IL‐10 expression, significant increase of CD3^+^ T cell proliferation in autologous co-culture assay, and increased production of IFN-γ (Th1 marker), and IL-4 (Th2 marker) compared to non-CTLA-4-silenced DCs	Anti-CTLA-4 shows promising anti-tumor effects via increase in inflammatory cytokines and T cell proliferation	Ghorbaninezhad et al. [53]
cDC1	IL-12	Zbtb46-DTR bone marrow chimeric mice with MC38 tumor	Anti-mouse PD-1	Mice lacking DCs failed to reject tumors in response to aPD-1, and mice with neutralized IL-12 failed to eject tumors in the presence of aPD-1	IL-12 production of cDC1s is crucial to anti-PD-1 therapy response	Garris et al*.* [55]
cDC1 (identified by CD141^+^)	cDC1 abundance	NSG-SGM3 mice with human CD34^+^ hematopoietic stem cells (HSCs) from umbilical cord blood transplanted with human melanoma cell line	Pembrolizumab (anti-PD-1 drug)	Enhancement and activation of CD14^+^ DCs increased anti-PD-1 efficacy in mice; intratumoral injections of CD141 led to reduced tumor growth in combination with anti-PD-1 treatment	cDC1s may be a critical indicator of response to ICB therapy	Lee et al. [56]
cDC2	CD5	MCA1956 tumors in DC specific CD5 knockout mice Versus control mice	Anti-mouse PD-1	DC specific CD5 knockout mice did not respond as efficiently and had fewer tumor rejections in anti-PD-1 therapy compared to control mice. Also, CD5^+^ DCs increased in tumors that were responsive to anti-PD-1	CD5 expression on cDC2s (the main subset expressing CD5) contributes to response to anti-PD-1	He et al. [57]
moDC (moDC2 subset)	Abundance	B16 and MC38 mouse models, scRNA seq data from patients with melanoma	Anti-mouse PD-1 for mouse experiments; nivolumab for patient samples	Tumors responsive to anti-PD-1 were found in MC38 mice, showing increased moDC2 and monocytes per tumor volume. Findings confirmed by single cell RNA-seq of human melanoma treated with anti-PD-1 also found responding tumors enriched for moDCs and increased TIL cytotoxicity	moDCs may be a critical indicator of response to ICB therapy	Schetters et al. [58]
moDC (indirect)	Indirect upregulation of moDC CXCL10 via RORyT agonist	LLC, B16F10 and MC38 mouse models	Anti-mouse PD-1	The combination of the RORyT agonist and anti-PD-1 inhibited tumor growth in all three mouse models more effectively than in mice treated with the agonist or anti-PD-1 alone	Highlights the importance of moDCs in antitumor immunity and also suggests targeting T cell features can improve response to ICB therapy via DCs	Xia et al. [59]
cDC1 (indirect)	Upstream Flt3/Flt3L signaling axis	B16 melanomas and TRAMP prostate adenocarcinomas	Anti-mouse CTLA-4	Administration of FLT3L-expressing Vaccinia virus in conjunction with anti-CTLA-4 improved outcomes in B16 and TRAMP mice	Combining these findings with evidence supporting upregulation of cDC1 abundance and function via Flt3, upregulation of Flt3/Flt3L signaling may enhance ICB therapy by indirect stimulation of cDC1s	Cueto et al. [62]
LAMP3^+^ mregDC (indirect)	Tht T cell program	B16-OVA + CD45.1^+^CD4^+^OT-II T cell (Tht model)—injeected C57Bl/6J mice	Anti-mouse PD-1	Observation of elevated levels of the ThT-I and II cell states in breast cancer lesions treated with pembrolizumb; OT-II-adoptively transferred mice treated with anti-PD-1 underwent significant tumor reduction, and both mThT cells and anti-PD-1 treatment were required for tumor response	Combining these findings with the observation that mregDCs are in close proximity to Tht cells and stimulate ThT cells via antigen presentation in in NSCLC lesions, mregDCs may have an indirect role in conferring response via ThT cell program in an anti-PD-1 setting	Cohen et al. [65]

Table columns list reference to the original paper in which study was conducted, the targeted DC subset and feature/marker of the DC investigated in the experiment, model used, immune checkpoint inhibitor in the form of antibody or FDA-approved drug, results and findings from the experiment, and implication of the findings in the context of ICB

**Table 2:** Significant findings of the impacts of anti-PD-1/PD-L1 and anti-CD47 combination on macrophages

Model	Treatment	Conclusions	Study
CAL27 and FaDu (head and neck squamous cell carcinoma) in murine model	Anti-PD-1	PD-1 blockade leads to reduction in CD47/SIRPɑ expression in myeloid cells	Yu et al. [70]
NJH29, NCI-H82, NCI-H69, NCI-H526 (small cell lung cancer) in murine model	Anti-CD47 and radiation therapy	Combination of anti-CD47 and radiation therapy significantly inhibits tumor growth and stimulates abscopal effect	Nishiga et al. [80]
D270 (malignant glioma) murine model	Anti-CD47	Anti-CD47 leads to increased phagocytic activity by both M1 and M2 macrophages	Zhang et al. [81]
Various colorectal carcinoma cell lines in murine model	RT and anti-CD47 and anti-PD-L1 combination	Triple combination leads to further reduction of tumor size in addition to reduction in abscopal tumors	Hsieh et al. [84]
SiHa and C33A (cervical cancer) in murine model	LSD1 inhibitor with anti-CD47/PD-L1	LSD1 correlated with CD47/PD-L1 expression, LSD1 inhibition with each of anti-CD47 and anti-PD-L1 inhibited tumor growth better than single blockade	Xu et al. [85]
ESCC in murine models and humans	Anti-CD47 and anti-PD-1 and anti-CTLA-4 combination	CD47 was associated with poor T cell infiltration, combination of anti-CD47, anti-PD-1, and anti-CTLA-4 results in smaller tumor size when compared to anti-CD47 and to anti-PD-1 and anti-CTLA-4 combination	Tao et al*.* [71]
Soft tissue sarcoma in vitro	Anti-CD47 and anti-PD-L1 combination	Anti-CD47/PD-1 combination leads to decrease in cytokine secretion when compared to anti-CD47 or anti-PD-1 individually	Ozaniak et al., 2022 [86]
CT26, MC38, and B16F10 (colorectal, colorectal, and melanoma, respectively) in murine model and cynomolgus monkey	Bispecific antibody with preferential binding to PD-L1 over CD47	BsAb shows improved survival rates over anti-CD47/PD-L1 and its combination and increased T and myeloid cell stimulation	Chen et al. [89]
Various lymphoma cell lines in murine model	Bispecific antibody with preferential binding to PD-L1 over CD47	BsAb shows smaller tumor sizes than when treated with either anti-PD-1 or anti-CD47 and increased T cell stimulation	Ke et al. [90]

Table columns list reference to the original paper in which study was conducted, model used, immune checkpoint inhibitor (and other treatment combinations), and results from the experiment

## Dendritic cells (DCs)

### Targeting immune checkpoints on DCs

Checkpoint inhibitors were developed with the notion of preventing T cell exhaustion by inhibiting the negative interaction between tumor cells and T cells, which is the last step of the cancer immunity cycle [5, 9, 28, 47] (discussed in our back-to-back review). However, myeloid cells also express PD-1 and PD-L1 and can prevent the recruitment of T cells to the TME even before they interact with cancer cells [40, 48–51]. While PD-1 and PD-L1 interaction usually contribute to an immunosuppressive environment by suppressing CD8^+^ T cell function and anti-tumor immunity, their presence is beneficial as they can be directly targeted by antibodies against them and are inhibited from their immunosuppressive function.

Several studies have shown that the expression of PD-L1 on DCs rather than on tumor cells may be more indicative of ICB response. Peng et al. [48] demonstrated the importance of PD-L1 expression on DCs in the observation of therapeutic effects of PD-L1 blockade therapy by showing higher PD-L1 expression on DCs than other cells in the TME and slower tumor growth in DC-conditional PD-L1 knockout mice compared to control mice. The knockout mice failed to respond to anti-PD-L1 with PD-L1 levels remaining the same on other cell types, suggesting that anti-PD-L1’s main target is PD-L1 on DCs. Specifically, the cDC1 subset was shown to play a crucial role in an effective response to anti-PD-L1. cDC1 upregulated PD-L1 after tumor antigen uptake and was dependent on IFN-γ and T cells in the TME. PD-L1 blockade was able to increase CD8^+^ T cells in the tumor, pointing to the recovered role of cDC1. Maier et al. [40] found similar findings to Peng et al*.* [48] but attributed PD-L1 upregulation to be on mregDCs derived from the cDC1 lineage and its upregulation to be dependent on the tyrosine kinase receptor AXL. It is possible that both papers describe the same subset, and more characterization of the DCs used in both experiments would be needed to confirm enrichment of the same pathways and molecules. Overall, given that PD-L1 on DCs mediate anti-PD-L1 effects, being able to modulate PD-L1 expression on DCs through the described pathways may greatly enhance anti-PD-L1 efficacy in naturally unresponsive tumors.

CTLA-4 is upregulated on activated T-cells and competes with CD28 to engage with CD80 or CD86 on APCs to inhibit T cell response [47]. Previous research attributed CTLA-4 on moDCs to decrease DC maturation and antigen presentation capability [52]. More recently, Ghorbaninezhad et al. [53] demonstrated that in an in vitro model, silencing CTLA-4 on monocyte-derived mature DC characterized by the expression of HLA-DR, CD40, CD86, and CD11c that were loaded with colorectal cancer cell lysate led to increased maturation and activation of DCs and enhanced T cell activation, proliferation, and cytokine production favoring an anti-tumor immune response. Given anti-CTLA-4 as a well-established ICB therapy, these findings raise a possibility of DCs mediating anti-CTLA-4 effects in vivo and validate the targeting of CTLA-4 on DCs to enhance ICB efficacy-in cancers.

PD-1 is also expressed on DCs [54] but has not been studied in an anti-PD-1 immunotherapy context to the extent that PD-L1 has in an anti-PD-L1 setting (as described above). It is possible that DCs confer anti-tumor immunity in an anti-PD-1 setting but not by direct engagement with PD-1 on DCs. Rather, anti-PD-1 seemed to trigger IL-12 production that was restricted to cDC1, but they did not express PD-1 at the transcript or protein level. Furthermore, IL-12 production was dependent on IFN-γ production by CD8^+^ T cells. These results may suggest that anti-PD-1 has a more T-cell-centric mechanism in contrast to anti-PD-L1, but its upstream or downstream effectiveness heavily relies on IL-12^+^ DCs [55].

Given that CTLA-4, PD-1, and LAG-3 exert inhibitory effects on DCs and T cells via distinct mechanisms of action, it was hypothesized that simultaneously blocking multiple signals could potentially amplify the anti-tumor response. Li et al. [42] describes several studies targeting multiple immune checkpoint molecules on DCs such as TIM-3, LAG-3, and CD47 in combination with PD-L1. mregDCs express the most TIM-3 out of DCs and macrophages, and given the definition of mregDCs, they may also highly express LAG-3 and CD47. The studies found higher levels of mregDCs’ capability in antigen uptake and communication with effector T cells upon combinatorial targeting of multiple immune checkpoints [42]. These findings stress the need for a systematic classification of mregDCs as they showcase properties with great therapeutic potential.

### DCs as cellular and molecular mediators of ICB response

While the expression of checkpoint molecules on DCs may offer a partial explanation to better response to ICB therapy, the mere presence of certain subsets of DCs and/or markers expressed on DCs may also positively contribute in an ICB setting. For example, in addition to cDC1 and mregDC expression of PD-L1 described above [40, 42, 48], Lee et al*.* [56] investigated the role of CD141^+^ cDC1 in advanced human melanoma and in a humanized mouse model. Using whole blood samples of advanced melanoma, patients who did not respond to anti-PD-1 and/or anti-CTLA-4 had decreased cDC1 number and function at every time point after treatment, whereas responding patients showed similar levels of cDC1 pre-treatment and at the time they showed clinical response. Nonresponders also showed lower plasma TNF-⍺ and IL-8 levels in the absence of toll-like receptor (TLR) stimulation and lower IFN-γ and IFN-β after TLR stimulation during treatment with immunotherapy. Using a humanized mouse model, evidence supported that increased numbers of cDC1 enhanced response to anti-PD-1 treatment. Although the study did not test the function of CD141, the main marker used to identify cDC1s, they investigated costimulatory markers and cytokines related to the subset (such as Flt3L and a TLR3 agonist to activate DCs) which may serve as an underlying explanation for the positive effects of a higher abundance of cDC1s. Another explanation may be related to the discovery by Gerhard et al*.* [21] of cDC1’s role in maintaining a proliferative set of tumor-antigen specific TCF1^+^ CD8^+^ T cells in tdLNs, where TCF1 is a transcription factor that drives T-cells’ response to ICB. It would be noteworthy to connect these findings in a single model to gain a comprehensive understanding of the role of cDC1s in different organs (blood system, lymphatic system and tumors) in response to ICB.

Recently, He et al*.* [57] identified the CD5 marker expressed on cDC2s, which correlated with greater survival and relapse-free survival in patients across several cancer types. They demonstrated that CD5 has a critical immunostimulatory function by increasing production of IFN-γ and TNF-⍺ by T cells, inducing antigen-specific memory T cells, and potentiating T cell effector function. Moreover, depletion of CD5-expressing DCs in tumor-bearing mice led to poorer response to anti-PD-1 than in control mice. Additionally, the frequency of CD5^+^ DCs increased in tumors that were responsive to anti-PD-1. The depletion of CD5 in DCs affected the expression of CD5 on CD4^+^ and CD8^+^ T cells, which affected tumor elimination in response to ICB therapy. CD5 depletion on T cells in murine models also negatively impacted the efficacy of ICB therapy, suggesting CD5 to have a broader presence and role that is not specific to DCs. Therefore, the positive correlation of CD5 on DCs and T cells seems to play an important role in the outcome of ICB therapy.

moDCs may also offer prognostic value in ICB therapy. Schetters et al. [58] found that moDCs were crucial in driving the increase of tumor-specific effector cells during anti-PD-1 treatment. moDCs were the most abundant APC in B16 melanoma and MC38 colorectal carcinoma (which are known to be ICB-insensitive and sensitive, respectively) mouse models and showed differentiation from monocytes to a phenotype expressing MHC I and II as well as co-stimulatory and co-inhibitory molecules. However, anti-PD-1 response was found only in MC38 mice, which had higher abundance of the moDC subset, moDC2 (a subset of moDCs the authors classified), and monocytes than the B16 mice before treatment. Specifically, the presence of moDC2 correlated with expanding tumor-infiltrating lymphocytes (TIL) in the anti-PD-1 treated MC38 mouse model, and scRNA-seq of human melanoma treated with anti-PD-1 confirmed a similar finding of responding tumors enriched for moDCs and increased TIL cytotoxicity. CD86 expression on moDCs (which was higher on moDC2 than moDC1) was suggested as a potential receptor to mediate PD-1 blockade efficacy via CD28 expression on T cells. Additionally, by using an agonist for CD40, which is highly expressed on moDCs in tumors, in combination with anti-PD-1, TIL expansion was also observed. CD40 is also highly expressed on mregDCs [42], suggesting that these targets may not be subset-specific. A more recent finding from Xia et al. [59] points to CXCL10 derived from moDCs to enhance CD8^+^ T cell migration in an anti-PD-1 setting. In summary, multiple features of moDCs may have valuable prognostic value in ICB therapy and would be promising targets for enhanced response.

pDCs have also been studied in the context of ICB. pDCs, although generally associated with poorer tumor response, may enhance cross-priming of CD8^+^ T cells in a cDC-dependent manner [41]. For example, a pDC vaccine given in combination with the anti-PD-1 drug pembrolizumab enhanced tumor antigen-specific CD8 T-cells in melanoma *ex-vivo* [60]. pDCs in cancer immunotherapy have been reviewed in Fu et al. [41].

### Upstream and downstream pathways enhancing ICB response via DCs

Given the evidence that supports DCs having a vital role in antitumor response in ICB treated tumors, it is also worth investigating pathways that affect or are affected by DC abundance and antitumor function in the TME. One such pathway is the Flt3/Flt3L signaling axis. Flt3, a well-known tyrosine kinase receptor, is crucial for regulating hematopoiesis [61] and was described by Cueto et al. [62] to have a main role in generating cDCs and pDCs. Specifically, it seemed that cDC1 function was upregulated by Flt3 ligand (Flt3L) in preclinical studies based on the proliferation of tumor-specific CD8^+^ T cells in tdLNs. Stimulating the Flt3/Flt3L axis with anti-CTLA-4 improved outcomes in the B16 melanoma and TRAMP prostate adenocarcinoma mouse models [63]. More results from targeting Flt3 are described in Cueto et al. [62] and Oba et al. [64], where tumors in mice treated with radiation were responsive to anti-PD-L1 therapy after stimulating the Flt3 pathway and TLR3/CD40, which indirectly increased the level of cDC1s in the TME. These results suggest promise for targeting the Flt3/Flt3L axis in tumor immunotherapy via cDC1 stimulation.

The study showing CXCL10-enhanced T-cell migration as mentioned in the previous section [59] pinpointed RORγT, a transcription factor of Th17 cells, as their main target. Using a small-molecule RORγT agonist, Xia et al. [59] found the agonist could stimulate Th17 cell differentiation and cytokine production in mouse models. The increased production of CXCL10 by moDCs was due to the upregulation of CCR6 and CCL20 by Th17 T cells, which promoted the migration of moDCs, leading to production of CXCL10. The introduction of the RORyT agonist also increased the efficacy of anti-PD-1 in tumor-bearing mice. Targeting RORyT also stimulated T-cell migration and infiltration, suggesting that targeting pathways more upstream may have an amplified effect that can influence multiple types of immune cells.

Another study by Cohen et al. [65] identified a specific T-cell program mediated by DCs in the TME that facilitated an antitumor response to anti-PD-1 treatment. The resulting T cells were termed T-helper tumor (Tht) cells, characterized by CXCL13^+^, PD-1^+^, and CD4^+^, and were present in NSCLC, melanoma, and breast cancers. In NSCLC tumors, mregDCs, which were identified by LAMP3^+^, were found to be in close proximity to Tht cells as well as CD3^+^ PD-1^+^ CD8^+^ T cells in TLSs in the TME. The observation of elevated levels of the Tht-I and Tht-II cell states in breast cancer lesions after anti-PD-1 (pembrolizumab) treatment suggested that Thts responded quickly and directly to anti-PD-1 ICB. Tht function was directly tested in mouse models with anti-PD-1, and the combination of the presence of Tht cells and anti-PD-1 resulted in tumor response. DCs expressing tumor antigens were responsible for the differentiation of the murine Tht cell state. This indicates that the presence of DCs are important in the success of anti-PD-1 ICB.

## Macrophages and monocytes

### Direct and indirect ICB effect on macrophages and MDSCs

PD-1 is also now known to be expressed on macrophages. Thus, anti-PD-1 has the potential to directly act on and reprogram macrophages in the TME. PD-1 expression in TAMs is associated with tumor progression in several cancers including lung cancer, gastric cancer, and colorectal cancer [49–51]. To study the effect of PD-1 signaling in myeloid cells, one study created PDCD1-floxed colon cancer mouse models with conditional PD-1 deletion in myeloid cells and T cells. The study found myeloid-specific PD-1 deletion was more effective than T cell-specific PD-1 deletion in restricting tumor growth, and as effective as global PD-1 deletion. To solidify the direct effect of the PD-1 antagonist on myeloid cells, the same study treated T and B cell deficient mice with anti-PD-1 and found a significant reduction in tumor growth [66].

The effect of PD-1/PD-L1 blockade is complex in MDSC, as studies have observed both reversal of MDSC-related immunosuppression leading to improved outcome and checkpoint resistance through MDSC recruitment. In vitro studies have seen anti-PD-L1 reversal of MDSC-mediated immunosuppression [67–69]. When co-cultured with T cells, MDSC expresses PD-L1 and directly interacts with the T cell PD-1 receptor. anti-PD-L1 blocks this interaction and prevents MDSC-mediated T cell suppression. A study on head and neck squamous cell carcinoma (HNSCC) found that PD-1 blockade significantly reduced tumor growth in the HNSCC mouse model, along with a significant reduction in MDSCs and TAMs in immune organs and tumors [70, 71]. On the other hand, a metastatic melanoma clinical trial found significant elevation of MDSC infiltration in post anti-PD-1 treatment patient biopsies [47]. The MDSC recruitment is likely an indirect result of IFN-γ influence, which is secreted by anti-PD-1 responding T cells. IFN-γ triggers NLRP3 inflammasome signaling cascade and ultimately leads to MDSC recruitment, thereby dampening the resulting antitumor immune response [72]. Interestingly, IL-1β, mostly secreted by infiltrating myeloid cells, plays a similar role by recruiting monocytes to the TME, which differentiate into macrophages under the influence of colony-stimulating factor-1 (CSF-1) [73]. This, in turn, decreases the proportion of CD11b^+^ DC proportion and thus IL-12 secretion, lessening its anti-tumor effects. This study highlighted a balance of macrophages and DCs in the TME in supporting antitumor immunity. These contradictory findings on MDSCs may be cancer-specific or context-dependent, which warrants further investigations in the future.

The cytokines secreted by anti-PD-1 stimulated T cells also influence the activation and thus function of macrophages [74]. Anti-PD-1 therapies have been shown to polarize macrophages toward the pro-inflammatory (M1-like) phenotype in the TME. Xiong et al. [75] have demonstrated that IFNγ secretion by T cells drives the phenotypic change, which enhances T cell responses. These findings have been supported by Gubin et al. [76], who observed a decrease in CX3CR1^+^ CD206^+^ macrophages and increase in iNOS^+^ macrophages in response to an increase in IFNγ. Macrophages have also been reported to express CXCL9, dependent on the production of IFNγ from T-cells [77]. CXCL9 is a ligand for CXCR3, and both CD8^+^ T-cell infiltration and the therapeutic efficacy of dual PD-1/CTLA-4 blockade were shown to be CXCR3 dependent using a mouse model of triple-negative breast cancer [77]. Similar findings were observed in a group of advanced melanoma patients who received combination anti-PD-1 and anti-CTLA-4. Responders of these patients contained higher CD16^+^ macrophages with upregulated gene expression of CXCL9, CXCL10 and CXCL11 [78]. Notably, the same CD16^+^ macrophage density difference between responders and non-responders was not observed in PD-1 monotherapy-treated patients. The distinct responses of macrophages to combination therapies as opposed to monotherapies underscore the need for more in-depth research in this area.

### CD47, an immune checkpoint specific for myeloid cells

CD47 is a surface protein found on many cells over the body and is known as a “don’t eat me” signal. The binding of CD47 on healthy body cells to SIRP-alpha on phagocytic cells, such as macrophages and monocytes, prevents the healthy cells from being attacked. In cancer, however, CD47 is overexpressed on tumor cells, allowing them to evade phagocytosis by the immune system. By targeting CD47 with antibodies and other agents that block the signal, phagocytic cells are able to “eat” cancer cells [79]. Nishiga et al. [80] showed that treating small cell lung cancer (SCLC) with radiotherapy and CD47 blockade leads to not only diminishing of the tumor at the local site but also off-set abscopal sites. Evidence suggests that ICB therapies targeting lymphoid cells rarely set off an abscopal response, though subsequent administration of radiation does increase the chance of a subsequent abscopal response. Additionally, it was suggested that CD47 blockade targets both pro-inflammatory (M1-like) and anti-inflammatory (M2-like) macrophage populations in the tumor microenvironment. Zhang et al. [81] also found anti-inflammatory macrophages to be responsive to CD47 blockade therapy. Additionally, the researchers found that cancer cells that became drug-resistant also became more vulnerable to macrophage-mediated cytotoxicity in response to anti-CD47 therapy. The downregulation of immunoinhibitory factors, such as B2M and CD73, on drug-resistant cancer cells could be a possible mechanism behind this effect. Therefore, combining anti-CD47 therapy with ICB therapies that target lymphocytes may lead to improved cancer treatment outcomes.

### Anti-CD47 and anti-PD-1/PD-L1 combination therapy

Various combination therapies, such as anti-PD-1 and anti-CTLA-4 combination therapy, have been shown to improve patient outcomes over a single ICB. These traditional combination therapies were designed to target multiple immune checkpoints on T cells, though as described above myeloid cells also express these checkpoints and may drive the response to ICB combinations. By targeting both PD-1 as well as SIRP-α on macrophages and monocytes, we reason that this combination may achieve better clinical outcomes by harnessing both the innate and adaptive arms of the immune system [82–84]. Myeloid cells, particularly macrophages, are critical in the therapeutic approach of combining CD47 and PD-1 blockade. In the context of checkpoint immunotherapies, most patients remain non-responsive, a problem that the CD47-SIRPα myeloid checkpoint blockade has shown potential in addressing particularly in hematologic malignancies. However, the expression of CD47 on peripheral blood has been a limiting factor in the selectivity and efficacy of anti-CD47 antibodies in solid tumors [85]. When investigating esophageal squamous cell carcinoma (ESCC), researchers found an increase in both PD-1/PD-L1 expression and CD47 expression. By inhibiting CD47 expression with ICB therapy, Tao et al. [71] reduced the tumor volume and increased tumor infiltration of T cells [71]. Furthermore, in colorectal cancer, when anti-CD47 and anti-PD-1 combination therapy is administered in addition to radiation therapy (RT), there was a further decrease in tumor volume and increase in percent survival, in both the irradiated and abscopal tumors [84]. Additionally, the triple therapy promoted APC cross-priming of CD8^+^ T cells, which was mainly driven by DCs, not macrophages [84]. However, not all cancer types show benefits to this combination therapy. A study in soft tissue sarcomas (STSs) did not find the expected synergistic qualities of the two treatments outlined in other studies [86]. The authors found that the combined use of anti-PD-1 and anti-CD47 therapy led to a significant decrease in cytokine production in the TME when compared to individual administration of each ICB therapy in STSs. This has been previously shown to correlate with degraded patient outcomes, potentially as a result of unexpected interactions between T cells, DCs, and macrophages [86–88].

Bispecific antibodies (BsAb) targeting multiple antigens have been explored as a possibility in recruiting both the innate and adaptive arms of the immune system against cancer. Studies by Chen et al. [89] and Ke et al. [90] have independently developed BsAbs to bind to both PD-1/PD-L1 and CD47, with a preference for PD-1/PD-L1. The use of the BsAbs were found to significantly improve outcomes, reducing tumor volumes when compared to either anti-CD47 or anti-PD-1/PD-L1 alone. RNA-sequencing analysis revealed that innate activation was contributed to mostly by the CD47/SIRPɑ axis. The treatment is additionally more specific to the TME and activated CD8^+^ T cells and increased their infiltration in the TME [89–92].

While anti-PD-1 and anti-CD47 combination therapy shows shrinking of tumor masses, further research is required to understand the changes in interactions between DC, T cell, and macrophage populations. Using spatial and single-cell sequencing techniques, we may be able to determine additional pathways to further increase the efficacy of this combination therapy.

## Discussion and future directions

In this review, we defined the broader class of myeloid cells and focused on DCs, and macrophages and their functions in the context of cancer immunity and ICB treatment. There is significant heterogeneity within each class, both in terms of the subsets present and their respective functions.

Among DCs, cDC1s and cDC1-like mregDCs seem to exhibit the most anti-tumorigenic properties by interacting with CD8^+^ T cells and being associated with a favorable prognosis in several cancers. Their abundance seems to be crucial in contributing to the success of ICB. cDC2s play a role in anti-tumor immunity by their interaction with CD4^+^ T cells, but cDC2-like mregDCs may adopt a more immunosuppressive function. moDCs share similar characteristics with cDC2s but lack the same capacity to stimulate T cells like cDCs; however, their presence also seems to benefit response to anti-PD-1 treatment. The suppression of pDC IFN-α production in the TME makes pDCs fall under the pro-tumor category, however, restoration of pDC function may confer benefit via other cells such as cDC1s. Much remains to be understood about DC subsets, including their diversity, spatial distribution, interactions with other immune cells, and varied responses in various contexts including different organs. Given the critical role of DCs in enhancing anti-tumor responses and the effects of immune checkpoint blockade (ICB), boosting DC function both directly and indirectly could intensify anti-tumor outcomes in cancers treated with ICB. Therefore, it's essential to examine how anti-PD-L1 and anti-CTLA-4 therapies directly affect DCs. Additionally, unraveling the intricate indirect effects of these therapies on various DC subsets is equally important. As many promising targets on or related to DCs exist, it would be interesting to know which targets may single-handedly provide the best outcome or provide synergistic effects when targeted in combination with ICB. Additionally, as most studies presented here observed effects in an anti-PD-1 setting, more tests should be conducted to see whether the impact of these features and pathways can be generalized to all ICB therapies, such as combination anti-PD-1 and anti-CTLA-4.

For monocytes and macrophages, they are known to influence tumor progression, promoting pro-inflammatory and anti-inflammatory responses in addition to aiding in the activation of CD8^+^ T cells. However, our understanding of the functional spectrum of macrophages and MDSCs is still incomplete. Similar to DCs, our knowledge about macrophage responses to different ICB therapies remains limited. To harness the anti-tumor potential of macrophages, studies used various CD47 ICB therapies which, when combined with ICB therapies that recruit the adaptive immune system, resulted in a robust anti-tumor effect. Despite the synergistic effect of the two ICB therapies that results in a more significant reduction in tumor sizes and greater survival rates, we do not know how the molecular mechanisms of the combination therapy compare to that of each individual ICB therapy. Single-cell analyses may reveal how macrophages, DCs, and CD8^+^ T cells respond to the combination ICB therapy and are especially helpful in determining whether the ICB results in an additive response or a previously unknown pathway is triggered.

Most studies reviewed here used mouse models at their base to perform genetic experiments with some also performing scRNA-seq analysis to identify the cell types and cellular states in the TME. Experiments solely using mouse models could benefit from single-cell and spatial transcriptomic studies by showing immune profiles before, during, and after ICB. However, both in vivo models and single-cell sequencing have their limitations. Animal models are usually developed where a gene or marker of interest is known a priori*,* otherwise experiments could be too costly and time intensive. The precision of single-cell technology is limited by its cross-sectional nature and the fluctuation of expression profiles at different time points [93], making it difficult to track the effect of new therapies. Recently, progress has been made in CRISPR screening assays where a construction of a sgRNA library can be added to a cell line or primary cells and transplanted into mice to observe effects on tumor growth. Library representation of the formed tumors is read by sequencing [94]. Using this technology in the context of ICB could prove as a more effective way to discover advantageous gene knockouts or overexpression. Additionally, combining CRISPR screening with single-cell readouts may lead to identification of master regulators controlling the dynamic development of immune subsets in an in vivo context.

Currently, many single-cell studies are still in the process of defining cell subsets using novel markers identified from scRNA-seq, which is especially the case for newly identified rare cellular states, such as mregDCs. As we witness progress in single-cell sequencing and lineage tracing technologies, concurrent advancements are also being made in their applications and analysis methodologies. These efforts would enable a standardized and robust protocol for comparing and integrating cell profiles across cohorts, which could greatly enhance the accuracy of DC and macrophage subset classification. Nonetheless, due to the modulating nature of the TME and the evolving and heterogeneous nature of tumors, a single consensus profile for each cell type may not be sufficient or meaningful to predict a patient's response. We anticipate the construction and modeling of a dynamic, tumor-immune ecosystem tailored to each individual tumor that could serve as a catalyst for the advancement of precision medicine within the field of immunotherapy. Another limitation to the study of DCs and macrophages in the TME is the feasibility of reproducing cellular states in vitro that express the same set of markers as in vivo and have the proper environment to maintain the state, as many subsets rely on the production of certain molecules in the TME and interactions with other cell types. Yet achieving an accurate in vitro replication of immune interactions stands as a crucial benchmark for computationally constructing the aforementioned ecosystem, and empowers more physiologically relevant CRISPR screenings.
